# Modern Views of Dysentery. The Serum Test

**Published:** 1903-06-20

**Authors:** 


### MODERN VIEWS OF DYSENTERY. THE SERUM TEST.

It has for some time been thought that dysentery, which represents one of the four great epidemic diseases of the world, is due to specific organisms. Of the organisms most generally recognised as associated with these diseases, the bacillus dysenteriæ of Shiga and other observers, and the amœbæ of Lambl, and afterwards of Councilman and Lafleur are perhaps the best known. It has been shown that each of these organisms is responsible for a particular and well-defined form of dysentery, so that the terms, bacillary dysentery, and amœbic dysentery, have come into general use. Bacillary dysentery is the common dysentery of India, and of other tropical regions. The bacillus is found in the evacuations as well as in the intestines, of which the sigmoid flexure of the colon and the rectum are most seriously affected. The mucosa and submucosa are severely inflamed, and the formation of very irregular ulcers situated on a generally uniformly, much thickened mucous membrane are characteristic. The appendix very often escapes, but the lower end of the ileum is often granular and swollen. Serious complications are uncommon, and large single abscess of the liver never occurs in this form of dysentery, the mortality from which is not excessive. Amœbic dysentery is comparatively much less common. The cæcum is the part of the intestine most seriously affected, and the appendix is often involved, and may be perforated. The lesions are characterised by raised oval ulcers projecting above the level of unaltered mucous membrane around them. In passing down the colon the ulcers become smaller and less numerous, until they are represented by minute pinhead areas, intensely injected, some of which show a yellowish gelatinous centre. Swarms of large amœbæ are to be found in these little areas, as well as in the bases of the ulcers in the cæcum and appendix. The amœbæ are also found in the evacuations. Amœbic dysentery runs a chronic course, is often latent, and though seldom fatal of itself, its complications are frequent and formidable. The most important complications are large abscess of the liver, or of the liver and lung, chronic peritonitis, acute perforative peritonitis, and retro-peritoneal abscess. Large abscess of the liver occurs as a complication as often as in one out of every four or five cases of amœbic dysentery. The pus is sterile in more than half the cases, and the abscess always contains the amœba dysenteriæ in its walls, and it can generally be found in the pus too. It is an important fact, moreover, that the lesions typical of amœbic dysentery are nearly always found in patients dying from large abscess of the liver, which never follows bacillary dysentery. It is therefore a matter of importance to differentiate clinically the common or bacillary form of dysentery from the rarer variety, amœbic dysentery, with its many and often fatal complications. The serum test, resembling Widal's, for enteric fever, has been applied to cases of dysentery. The test is dependent upon the properties of agglutination of the bacillus dysenteriæ, which the blood of patients suffering from common or bacillary dysentery possesses. On the other hand, the blood taken from patients with amœbic dysentery possesses no power of agglutination of the bacilli. Rogers [1] describes the results of observations in 17 cases in which this test was applied and proved correct. In 16 clumping of bacilli took place, and these all showed the other signs typical of bacillary dysentery, the lesions and bacillus being found in four of the cases which were submitted to a post-mortem investigation. In the single case in which the test was negative, it was also shown both clinically and at the autopsy that the disease was genuine amœbic dysentery. Should these observations be confirmed by others on a larger series of cases, the application of the serum test may prove as valuable in differentiating the varieties of dysentery as it has been in the classification of other diseases.

[1] Indian Medical Gazette, Feb., 1903.

